# Antithrombin Regulates Matriptase Activity Involved in Plasmin Generation, Syndecan Shedding, and HGF Activation in Keratinocytes

**DOI:** 10.1371/journal.pone.0062826

**Published:** 2013-05-13

**Authors:** Ya-Wen Chen, Zhenghong Xu, Adrienne N. H. Baksh, Jehng-Kang Wang, Chiu-Yuan Chen, Richard Swanson, Steve T. Olson, Hiroaki Kataoka, Michael D. Johnson, Chen-Yong Lin

**Affiliations:** 1 Greenebaum Cancer Center, University of Maryland, Baltimore, Maryland, United States of America; 2 Graduate Program in Life Science, University of Maryland, Baltimore, Maryland, United States of America; 3 Department of Medicine, University of Illinois in Chicago, Chicago, Illinois, United States of America; 4 Department of Biochemistry, National Defense Medical Center, Taipei, Taiwan; 5 Graduate Institute of Natural Healing Sciences, Nanhua University, Chiayi, Taiwan; 6 Center for Molecular Biology of Oral Diseases, University of Illinois in Chicago, Chicago, Illinois, United States of America; 7 Department of Pathology, Faculty of Medicine, University of Miyazaki, Miyazaki, Japan; 8 Lombardi Cancer Center, Georgetown University, Washington, D.C., United States of America; Institute of Molecular and Cell Biology, Singapore

## Abstract

Matriptase, a membrane-associated serine protease, plays an essential role in epidermal barrier function through activation of the glycosylphosphatidylinositol (GPI)-anchored serine protease prostasin. The matriptase-prostasin proteolytic cascade is tightly regulated by hepatocyte growth factor activator inhibitor (HAI)-1 such that matriptase autoactivation and prostasin activation occur simultaneously and are followed immediately by the inhibition of both enzymes by HAI-1. However, the mechanisms whereby matriptase acts on extracellular substrates remain elusive. Here we report that some active matriptase can escape HAI-1 inhibition by being rapidly shed from the cell surface. In the pericellular environment, shed active matriptase is able to activate hepatocyte growth factor (HGF), accelerate plasminogen activation, and shed syndecan 1. The amount of active matriptase shed is inversely correlated with the amount of antithrombin (AT) bound to the surface of the keratinocytes. Binding of AT to the surface of keratinocytes is dependent on a functional heparin binding site, Lys-125, and that the N-glycosylation site Asn-135 be unglycosylated. This suggests that β-AT, and not α-AT, is responsible for regulation of pericellular matriptase activity in keratinocytes. Keratinocytes appear to rely on AT to regulate the level of pericellular active matriptase much more than breast and prostate epithelial cells in which AT regulation of matriptase activity occurs at much lower levels than keratinocytes. These results suggest that keratinocytes employ two distinct serine protease inhibitors to control the activation and processing of two different sets of matriptase substrates leading to different biological events: 1) HAI-1 for prostasin activation/inhibition, and 2) AT for the pericellular proteolysis involved in HGF activation, accelerating plasminogen activation, and shedding of syndecans.

## Introduction

Epidermal differentiation is a carefully controlled process that generates a functional epidermal layer providing the critical barrier function of the skin [1], [2]. The process involves significant pericellular proteolysis for the progressive remodeling of cell morphology and tissue structure and must be regulated in a precisely controlled manner [3], [4]. Several genetic disorders that result in skin pathology have been linked to defects in proteolysis. Among the many proteases and protease inhibitors that are involved in skin functions, matriptase, prostasin, and HAI-1 have been shown to be functionally linked and form a tightly controlled protease/inhibitor network. Matriptase, a type II transmembrane serine protease (TTSP), functions as an initiator protease that undergoes autoactivation to convert matriptase zymogen to active matriptase [5]. Matriptase zymogen activation is an early event in epidermal differentiation [6]. Increased matriptase zymogen activation has been previously shown to be associated with various human skin diseases and may result from the oxidative environment associated with the inflammation, or acidification of the extracellular milieu associated with many pathologic states, since matriptase activation is induced in cells exposed to H_2_O_2_ or a mildly acidic environment [7], [8]. Matriptase zymogen activation and its control are, therefore, important physiological processes in the skin. Prostasin, a GPI-anchored serine protease appears to be the sole downstream substrate responsible for the epidermal defects associated with matriptase ablation in mice [9]. A remarkable feature of regulation of this serine protease cascade is that both proteases are under extremely tight control by HAI-1 [6]. HAI-1, an integral membrane, Kunitz-type inhibitor, is co-expressed and co-localized with matriptase with a HAI-1:matriptase protein ratio of more than 10∶1 in the majority of epithelial and carcinoma cells [10]. Interestingly, HAI-1 is required for normal matriptase synthesis and intracellular trafficking from the endoplasmic reticulum [11]. Furthermore, HAI-1 appears to participate in matriptase autoactivation [5]. As a consequence, active matriptase is inhibited by HAI-1 as rapidly as it is generated [8], as if both matriptase activation and inhibition take place at essentially the same time. Remarkably, in spite of having such a short life span, active matriptase is still able to activate prostasin [6]. The unusually tight linkage of the three key players of the protease network is consistent with the similar epidermal defects observed in their respective knockout mice [12]–[14].

In addition to prostasin, matriptase is also involved in the activation of urokinase plasminogen activator (uPA) and hepatocyte growth factor (HGF) [15], [16]. HGF activation by matriptase and subsequent induction of cMET pathway signaling is likely the mechanism responsible, at least in part, for the development of spontaneous squamous cell carcinomas in matriptase transgenic mice [17]. The expression of matriptase in THP-1 monocytes is thought to represent a plasmin-independent mechanism for uPA activation and to contribute to the shortened lag phase of plasmin generation [18]. The uPA/plasmin system has been implicated in the amplification of psoriasis-form skin inflammation [19] and the adhesion and migration of leukocytes during their recruitment from the circulation to extravascular sites of inflammation [20]. In addition, mice with *Plg* (plasminogen) and *Plau* (uPA) deficiencies have been shown to have diminished keratinocyte migration, and delayed re-epithelialization [21]–[23]. In contrast to prostasin that is co-expressed and co-localized with matriptase on the plasma membrane of keratinocytes, both uPA and HGF are proteins secreted either by keratinocytes or by stromal cells. Despite the growing evidence for the role of matriptase in the activation of uPA and HGF, there has been no tangible evidence for the presence of free active matriptase, produced by matriptase-expressing epithelial cells, that survives in un-complexed form long enough to activate these extracellular substrates [10]. The discovery of active matriptase with a prolonged active life would provide critical evidence for the role of matriptase in the activation of these extracellular substrates. In the current study, we demonstrated that a proportion of the active matriptase made by human keratinocytes can escape inhibition by HAI-1 through rapid shedding into the extracellular milieu during the process of matriptase activation. By exploring the mechanisms governing matriptase shedding and characterizing the substrates processed by matriptase in this context, our studies reveal that keratinocytes appear to have evolved two distinct inhibitory mechanisms to control the two different matriptase functions: 1) a mechanism involving the integral membrane, Kunitz-type inhibitor HAI-1 for the regulation of prostasin activation and 2) a mechanism involving the heparin sulfate proteoglycan (HSPG)-bound serpin-type inhibitor AT to regulate its role in the activation of uPA and HGF and the shedding of HSPGs.

## Materials and Methods

### Reagents

Horseradish peroxidase (HRP)-conjugated secondary antibodies were purchased from Kirkegaard & Perry Laboratories (Gaithersburg, MD), Western Lightning Chemiluminescence Reagent Plus was purchased from PerkinElmer Life Sciences (Waltham, MA). Nitrocellulose membrane was purchased from Pall Corp. (Pensacola, FL). Boc-Gln-Ala-Arg-AMC was purchased from Enzo Life Sciences (Farmingdale, NY) and Boc-Val-Leu-Lys-AMC was obtained from Bachem Americas, Inc. (Torrance, CA). Dithiothreitol (DTT) was purchased from Sigma-Aldrich (St. Louis, MD). ProtoBlue Safe was purchased from National Diagnostics (Atlanta, GE). Dulbecco's phosphate-buffered saline (DPBS) was obtained from Mediatech Inc. (Manassas, VA). Pro-HGF was prepared as described previously [24].

### Antibodies

Three types of antibodies were used to detect matriptase. The mouse monoclonal antibody (mAb) M24 and rat mAb 21-9 recognize both latent and activated forms of matriptase [6], [8], [25], [26]; the rabbit polyclonal antibody matriptase/ST14 (Bethyl, Montgomery, TX) recognizes an epitope present in the serine protease domain of matriptase and is, therefore, able to detect the matriptase/serpin complex after reducing and heating the samples. Another mAb M69 recognizes an epitope only present on activated matriptase and is able to distinguish activated matriptase from latent matriptase [27], [28]. HAI-1 was detected using the HAI-1 mAb M19 [25]. HGF protein was detected using a goat polyclonal antibody (Santa Cruz, Santa Cruz, CA) that recognizes the beta subunit of HGF. AT was detected using a goat polyclonal antibody (R&D, anti-serpin C1, Minneapolis, MN). Syndecan-1 was determined by a goat polyclonal antibody (R&D, Minneapolis, MN).

### Cell cultures

HaCaT cells (CLS Cell Lines Service GmbH, Eppelheim Germany) were maintained in Dulbecco's Modified Eagle Medium (DMEM) supplemented with 10% fetal bovine serum (FBS, heat-inactivated), 100 units/ml penicillin, and 100 µg/ml streptomycin. 184 A1N4 cells (a gift from M. R. Stampfer, UC Berkeley) [29] were maintained in DMEM/F12 with 0.5% FBS, 5 µg/ml of hydrocortisone (Sigma), 10 ng/ml of recombinant human epidermal growth factor (Promega), 5 µg/ml insulin, 100 units/ml penicillin, and 100 µg/ml streptomycin. RWPE1 cells (ATCC, Manassas, Virginia, USA) were maintained in RPMI 1640 with 10% FBS, 100 units/ml penicillin, and 100 µg/ml streptomycin. Human primary keratinocytes were maintained in keratinocyte-SFM (Invitrogen) supplemented with bovine pituitary extract (20–30 µg/ml), recombinant epidermal growth factor (rEGF, 0.1–0.2 ng/ml), 100 units/ml penicillin, and 100 µg/ml streptomycin.

### Acid-induced matriptase activation

HaCaT cells were washed with DPBS and then incubated either in DPBS, as a control, or 150 mM phosphate buffer, pH 6.0 for 30 minutes at room temperature, as described previously [6]. To assess the materials shed from the cells during incubation, the buffer was collected and concentrated for immunoblot analysis or matriptase activity assay. Cells were harvested and lysed in 1% Triton X-100 in DPBS and subjected to immunoblot analyses. The lysis buffer also contained 1 mM 5,5′-dithiobis-(2-nitrobenzoic acid) (DTNB) to prevent the cleavage of a disulfide bond that links the serine protease domain and the non-catalytic domain of matriptase [30].

### Gelatin zymography

For gelatin zymography, protein samples were incubated with 5× sodium dodecyl sulfate (SDS) sample buffer containing no reducing agent and incubated at room temperature for 5 min. The proteins were resolved by SDS-polyacrylamide gel electrophoresis (SDS-PAGE) using a 7.5% polyacrylamide gel containing 1 mg/ml gelatin. The gelatin gels were washed with DPBS containing 2.5% Triton X-100 to remove the SDS and incubated in Tris buffer pH 8.0 at 37°C overnight. The gels were stained by ProtoBlue Safe.

### Activation of pro-HGF

Five nanograms of pro-HGF were mixed with increasing amounts of active matriptase (0–400 pM). The mixture was incubated in a 96-well plate in the presence or absence of pre-seeded HaCaT cells at 37°C for 30 min. The samples of the supernatants were then analyzed by immunoblot for the cleavage of pro-HGF by matriptase using a HGF antibody.

### Purification of active matriptase

Acid-induced matriptase activation was performed using HaCaT cells and the conditioned buffer was collected and concentrated. The 70-kDa free active matriptase was purified by immunodepletion to remove the matriptase/HAI-1 complex using immobilized HAI-1 mAb M19, followed by immunoaffinity chromatography using immobilized matriptase mAb M69. The affinity columns used were pre-equilibrated with DPBS and eluted with 0.1 M glycine buffer pH 3.6. The eluents were immediately neutralized with 2 M Trizma base to pH 7.5. The concentration of active matriptase was determined by active site titration as previously described [31].

### Immunoblot

The protein extracts were mixed with 5× SDS loading buffer in the absence or presence of reducing agents and incubated either at room temperature or at 95°C for 5 min. Protein samples were resolved by 7.5% SDS-PAGE and transferred to nitrocellulose membranes. The membranes were probed with the desired antibodies and an HRP conjugated secondary antibody, followed by signal detection with Western Lightning Chemiluminescence Reagent Plus.

### Purification and identification of 110-kDa matriptase complex

The 110-kDa matriptase complex was generated concurrent with the activation of matriptase in HaCaT cells by treating the cells with phosphate buffer, pH 6.0 for 30 min at room temperature. The conditioned buffer was collected and concentrated. The 110-kDa matriptase complex was purified by sizing chromatography using HPLC (Beckman) with a Biosuite 250 sizing column at a flow rate of 2 ml/min, immunodepletion to remove the matriptase/HAI-1 complex using immobilized HAI-1 mAb M19, followed by immunoaffinity chromatography using immobilized matriptase mAb 21-9 to capture the 110-kDa matriptase complex. Both immunodepletion and immunopurification were previously described [6], [10]. The 110-kDa complexes and other bound proteins were eluted from the mAb 21-9 immunoaffinity column by 0.1 M glycine buffer, pH 2.4 and immediately neutralized by 2 M Trizama base. The eluate was resolved by SDS-PAGE and the protein bands were stained with ProtoBlue Safe. The two protein bands around 110-kDa were sliced from the gel, subjected to in-gel digestion with trypsin, and analyzed by liquid chromatography/mass spectrometry (LS/MS) using a service provided by Prottech Inc. (Norristown, PA).

### Protease activity assay

The matriptase or plasmin activity was assessed by a fluorogenic assay measuring **7-Amino-4-methylcoumarin** (AMC) release from synthetic substrates by the proteases. For matriptase activity, the conditioned buffer was concentrated up to 100 fold using Amicon Ultra-4 centrifugal filter units (Millipore, Billerica, MA). The assay was conducted in a total volume of 200 microliter which contained 5 µl of the concentrated samples, 5 µl of a 5 mM stock of the substrate (Boc-Gln-Ala-Arg-AMC), and 190 µl of 100 mM Tris-HCl (pH 8.5) containing 100 µg/ml bovine serum albumin. For plasmin activity assays, 10^4^ primary keratinocytes were seeded per well in 96-well plates. The next day, the cells were washed and incubated with DPBS, as a control, or the pH 6.0 buffer to induce matriptase activation. The contents of the wells were then adjusted to pH 7.5 by adding 5 ul of 2 M Trizma base. 50 nM plasminogen and 5 µl of a 5 mM solution of the substrate (Boc-Val-Leu-Lys-AMC) were added to the cells which were then incubated at 37°C. For both assays, the released fluorescence resulting from hydrolysis of the peptide substrates was measured using a fluorescent spectrophotometer (Bechman, DTX 880) with excitation at 360 nm and emission at 480 nm.

### AT binding assay

HaCaT cells were incubated in serum free basal medium for 30 min in a CO_2_ incubator at 37°C to remove bovine AT from the cells. Three preparations of AT at a concentration of 230 nM were then added to the cells and incubated for another 5 min in a CO_2_ incubator. The cells were then washed 3 times with DPBS and lysed. The levels of AT were assessed by immunoblot.

### Syndecan-1 shedding

HaCaT cells were washed with DPBS and incubated with increasing amount of active matriptase in basal medium at 37°C in CO_2_ incubator for 30 min. After incubation, the conditioned media were collected and analyzed for the amount of syndecan-1 shedding using a dot blot assay, as described by Bernfiled and colleagues [32].

## Results

### Active matriptase is either rapidly inhibited by HAI-1 or shed from keratinocytes

**Figure 1:**
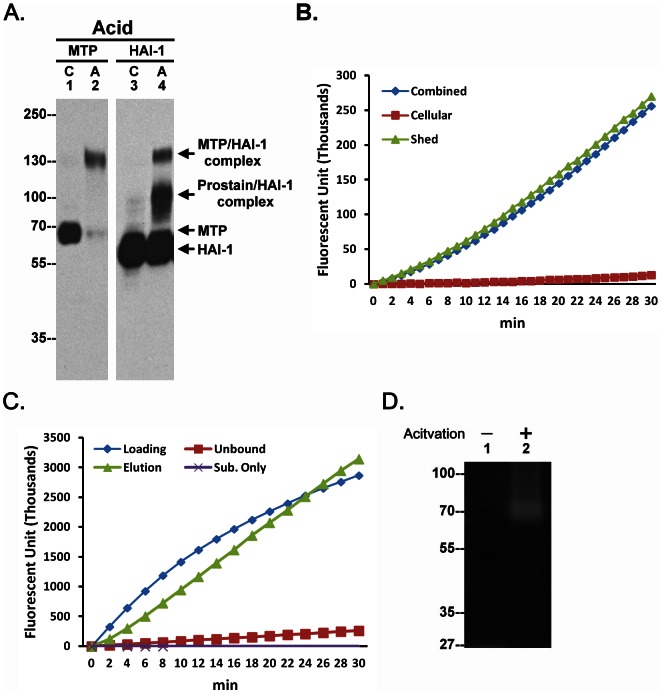
Shedding of active matriptase by human keratinocyte. *A.* Human keratinocyte HaCaT cells were incubated either with PBS (C) as a control or a pH 6.0 buffer (Acid, A) to induce activation of matriptase. Cell lysates were analyzed by immunoblot for matriptase (MTP) or HAI-1 (HAI-1). *B.* HaCaT cells were incubated with a pH 6.0 buffer to induce matriptase activation. The cells and the conditioned buffer together (Combined), the cells alone (Cellular) and the conditioned buffer alone (Shed) were analyzed for matriptase activity using a matriptase synthetic fluorescent substrate, Boc-Gln-Ala-Arg-AMC. Data are representative of four independent experiments done under similar conditions. *C.* The conditioned buffer was subjected to immunoprecipitation with the activated matriptase mAb M69. The loading control (Loading), the unbound fraction (Unbound), and the eluent (Elution) were assayed for matriptase activity using the substrate, Boc-Gln-Ala-Arg-AMC. Data are representative of three independent experiments done under similar conditions. *D.* HaCaT cells were incubated with DPBS as control or a pH 6.0 buffer to induce matriptase activation. The conditioned buffer was collected and subjected to gelatin zymography.

When keratinocytes were induced to activate matriptase by extracellular acidosis, 70-kDa matriptase zymogen was converted into a 120-kDa (activated) matriptase-HAI-1 complex (Fig. 1A, Acid, comparing lane 1 with lane 2). Prostasin, a matriptase substrate, was also activated and formed a 100-kDa prostasin-HAI-1 complex that can be detected using the HAI-1 mAb (Fig. 1A, Acid, lane 4). This is consistent with our previous study in which we demonstrated the unusually tight control and rapid kinetics (minutes) of the matriptase-prostasin proteolytic cascade which features simultaneous activation of matriptase and prostasin, followed by immediate HAI-1-mediated inhibition of both proteases [6].

In spite of the rapid inhibition of matriptase by HAI-1 that was observed under these conditions, the addition of fluorogenic substrates to the culture system revealed the presence of strong proteolytic activity (Fig. 1B, Combined). Interestingly, assays conducted on the cells and the conditioned buffer separately demonstrated that this proteolytic activity was present only in the conditioned buffer and was not associated with the cells (Fig. 1B). Immunodepletion of the conditioned buffer using a matriptase mAb removed the proteolytic activity from the buffer which could be then be recovered from the beads, confirming that the proteolytic activity was due to the presence of active matriptase shed into the buffer (Fig. 1C). The presence of active matriptase in the conditioned buffer was further confirmed by gelatin zymography (Fig. 1D).

### Pericellular active matriptase can activate pro-HGF and accelerate plasminogen activation

**Figure 2:**
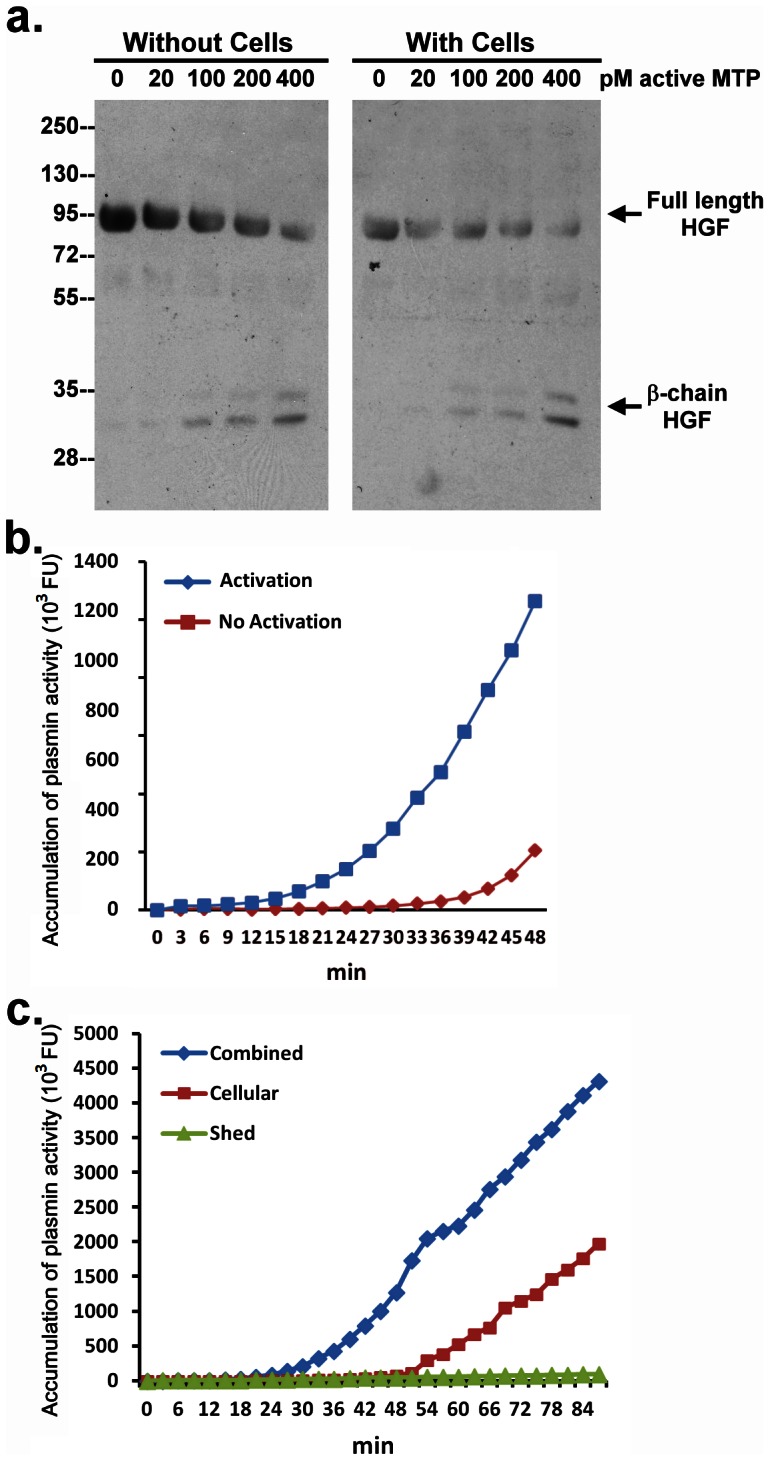
Activation of pro-HGF and acceleration of plasminogen activation by active matriptase shed from human keratinocytes. *a.* Pro-HGF was incubated with increasing amounts of active matriptase, as indicated, either with or without HaCaT cells at 37°C for 30 min. Samples were analyzed by immunoblot for HGF cleavage using an antibody directed against the beta subunit of HGF. *b.* Primary human keratinocytes were incubated with DPBS (No Activation) or a pH 6.0 buffer (Activation) to induce activation of matriptase, after which the buffer on the cells was adjusted to pH 7.5. Plasminogen (50 nM) was then added to the cells and the generation of plasmin was monitored by the cleavage of a plasmin synthetic fluorescent substrate, Boc-Val-Leu-Lys-AMC. Data are representative of three independent experiments done under similar conditions. *c.* Primary human keratinocytes were incubated with a pH 6.0 buffer to induce matriptase activation, followed by buffering to pH 7.5. Plasminogen (50 nM) was then added to the cells in the presence of the shed fractions (combined) or in the absence of the shed fractions (Cellular) or the shed fraction alone (Shed). Generation of plasmin was monitored by the cleavage of Boc-Val-Leu-Lys-AMC. Data are representative of four independent experiments done under similar conditions.

We next investigated whether the shed active matriptase could activate and process substrates in the pericellular environment in which cell surface HAI-1 might be expected to suppress the activity of shed active matriptase. Incubation of pro-HGF in solution with increasing concentrations of active matriptase resulted in the rapid conversion of single-chain pro-HGF into the two-chain active form of HGF in a dose dependent manner (Fig. 2a, left panel). Similar effects were also observed when both active matriptase and pro-HGF were incubated with HaCaT keratinocytes (Fig. 2a, right panel). These data suggest that in spite of the abundance of HAI-1 on the cell surface, active matriptase is able to activate pro-HGF with similar potency compared to that in the absence of cell-associated HAI-1.

Since matriptase has been identified as an activator of pro-uPA, we next tested whether pericellular active matriptase can enhance plasminogen activation. Due to the high levels of active plasminogen activator(s) (PA) present in HaCaT cells, we used primary human keratinocytes for this experiment. Plasminogen activation commonly features a lag phase followed by a burst of plasmin generation: a phenomenon known as “reciprocal zymogen activation” [18]. In order to test whether active matriptase in the pericellular environment might impact plasminogen activation, matriptase activation was induced in the primary human keratinocytes by exposing them to pH 6.0 buffer (or PBS as a control). After 30 minutes the pH of the buffer was adjusted to pH 7.5 and plasminogen and a fluorogenic plasmin substrate were added. As shown in Fig. 2b, it required almost 40 min for the activation of plasminogen to begin to take off in the absence of active matriptase (PBS treated cells) (Fig. 2b, No Activation). In contrast, in the presence of active matriptase (pH 6 treated cells), the lag phase was shortened by about 20 minutes (Fig. 2b, Activation).

Since we had shown that the free active matriptase activity is shed into the buffer, and is not associated with the cells (Fig 1B), we next examined the ability of the conditioned buffer to accelerate plasmin generation. When the shed active matriptase was separated from the cells and incubated with plasminogen, it failed to generate plasmin (Fig. 2c Shed). These data suggest that active matriptase cannot mediate plasmin generation in solution. Interestingly, the lag phase to plasmin generation was increased, occurring at around 54 min (vs 24 minutes) after plasminogen was added to the post-activation keratinocytes from which the conditioned buffer had been removed (Fig. 2c, Cellular). The combination of the cells and shed active matriptase significantly accelerate plasminogen activation (Fig. 2c, Combined). These intriguing data suggest that matriptase-mediated acceleration of plasminogen activation seems to require the presence of both activated matriptase shed into the medium and the keratinocytes, leading us to hypothesize that this event occurs at the cell surface. Active matriptase in solution is not a good activator for plasminogen, and the cells alone do not result in efficient plasmin generation, but active matriptase can accelerate plasmin generation when combined with the cells. This suggests that plasminogen activation occurs at the cell surface, either mediated directly by matriptase activity, or indirectly by matriptase-mediated activation of a cell-surface plasminogen activator. The latter mechanism is reminiscent of what has been suggested in monocytes in which the binding of urokinase plasminogen activator to its cell surface receptor is involved in plasmin generation [18].

### AT is an important matriptase inhibitor in human keratinocytes

**Figure 3:**
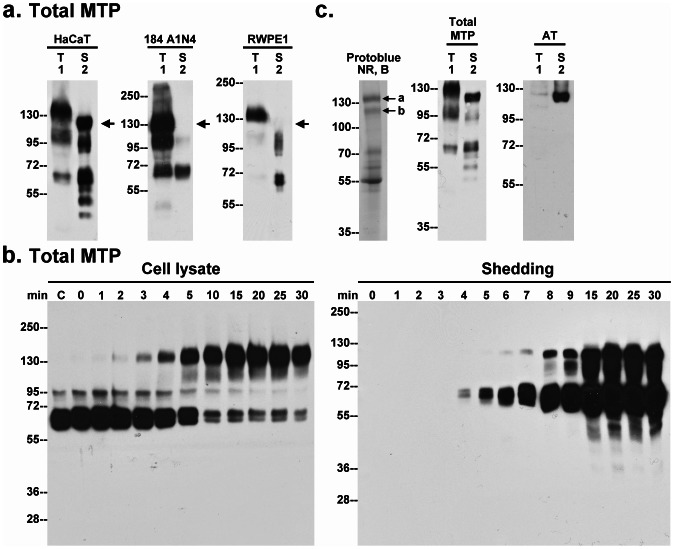
Identification of AT as a component of the novel 110-kDa matriptase complex. *a.* Human keratinocytes (HaCaT), mammary (184 A1N4) and prostate (RWPE1) epithelial cells were incubated with a pH 6.0 buffer to induce matriptase activation. The conditioned buffers (S) and the total cell lysates (T) were analyzed by immunoblot for total matriptase. Arrow indicates the 110-kDa matriptase complex. *b.* HaCaT cells were incubated with a pH 6.0 buffer for the indicated times (minutes). The cell lysates and the conditioned buffers were subjected to immunoblot analyses for total matriptase. *c.* Left panel: Purified 110-kDa matriptase complex was analyzed by SDS-PAGE under non-reducing and boiled conditions (NR, B). The protein bands were visualized by staining with ProtoBlue Safe. The protein bands *a* and *b*, as indicated, were subjected to protein identification by MS/MS (see Fig. S2 for sequence data). Middle and right panels: HaCaT cells, pretreated with human serum, were induced to activate matriptase by pH 6.0 treatment. Cell lysates and the concentrated conditioned buffers were subjected to immunoblot analyses by immunoblot for total matriptase (Total MTP) and AT (AT).

During the course of our studies we noticed the presence of a 110-kDa matriptase complex in the conditioned buffer from pH 6 treated HaCaT cells (Fig. 3a, HaCaT, arrow). This particularly caught our eye since we had not seen a similar band in other epithelial systems. When robust matriptase activation is induced in mammary (184 A1N4) and prostate (RWPE1) epithelial cells which results in the appearance of the expected 120-kDa matriptase-HAI-1 complex in cell lysates (Fig. 3a, lanes 1), the 110-kDa matriptase species was not detected in the conditioned buffer from these two cell lines. These data suggest that shedding of the 110-kDa matriptase species may be a keratinocyte-associated event.

The shedding of the 110-kDa species apparently closely follows the kinetics of matriptase activation: Matriptase activation begins at two minutes post acid-induction with the appearance of the matriptase-HAI-1 complex, and reaches a plateau about 10 minutes later (Fig. 3b, Cell lysate); the shedding of the 110-kDa species was detected at 6 min and accumulates to significant levels a few minutes after the completion of matriptase activation (Fig. 3b, Shedding).

Analysis of the 110-kDa matriptase complex showed that it was most likely a matriptase-serpin complex based on its resistance to heat treatment and the observation that the matriptase serine protease domain appears to be covalently bound to the binding protein (Fig. S1). In order to identify the putative serpin inhibitor involved, the complex was isolated using a combination of sizing chromatography, immunodepletion to remove matriptase-HAI-1 complex using immobilized HAI-1 mAb M19, and immunoaffinity chromatography using immobilized matriptase mAb 21-9. Following these procedures, the purified proteins were resolved by SDS-PAGE under non-reducing and boiled conditions. Several protein bands were observed with two protein bands, close to the expected 110-kDa molecular size, likely to be the matriptase complex (Fig. 3c, left panel, a and *b*). In-gel trypsin digestion followed by MS-based protein identification of the components of the two protein bands revealed that they were indeed comprised of matriptase, as expected, and the serpin inhibitor antithrombin (AT). The sequence information from the analysis is shown in the supplemental data (Fig. S2) and suggests that the two complex bands represent intact and cleaved forms of the complex. In order to validate this identification, HaCaT cells were incubated with human serum followed by the induction of matriptase activation by exposure to pH 6 buffer. Immunoblot analysis of the cell lysate and the shed fractions using matriptase mAb (Fig. 3c, Total MTP) and an AT antibody (Fig. 3c, AT) demonstrated that the 110-kDa species was recogized by both antibodies. Interestingly, the matriptase-AT complex was also detected at very low level in the cell lysate (Fig. 3c, lanes 1), suggesting that the vast majority of matriptase-AT complexes have been rapidly shed from the cell surface upon complex formation.

### Binidng of AT to keratinocyte cell surfaces is dependant on its heparin binding affinity and affects the levels of active matriptase shed into the extracellular milieu

**Figure 4:**
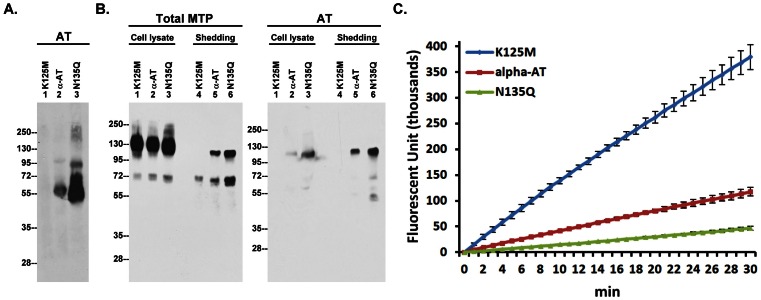
The shedding of active matriptase is inversely correlated with the levels of membrane-bound AT. *A*. HaCaT cells were incubated with the three AT preparations, as indicated, at 37°C for 5 min. The cellular retention of AT were analyzed by immunoblot analyses using an AT antibody. *B*. The AT-pretreated cells were then induced to activate matriptase by a pH 6.0 buffer. The cell lysates and the conditioned buffers were analyzed for matriptase species and AT species, as indicated. *C*. Matriptase activity in the conditioned buffer was also assessed by the cleavage of Boc-Gln-Ala-Arg-AMC. Data was done in triplicate and is a representative example of three independent experiments.

In order to effectively inactivate matriptase in cells, AT must first binds to the cell surface, likely via heparan sulfate proteoglycans (HSPGs) [31], [33]. The binding of AT to heparin or heparan sulfate is known to be mediated by a two-step mechanism in which the Lys-125 residue of AT provides the initial binding to the charateristic pentasaccharide in heparin [34], [35] with binding further strengthened by the ensuing conformational changes in AT [36]. Three AT preparations were used to invetstigate the role of heparin-binding affinity in cellular retention and matriptase inhibition: 1) AT-K125M, a mutant bearing a point mutation at the initial heparin binding residue Lys-125, 2) α-AT, purified from human serum, the major form which has oligosaccharide side chains occupying all four glycosylation sites, and 3) AT-N135Q, a β-AT analog with a point mutation at Asn-135. β-AT is a minor species in serum in which the Asn-135 N-glycosylation site is unoccupied and which exhibits higher binding affinity for heparin/heparan sulfate than α-AT. HaCaT cells were washed to remove bovine AT from the tissue culture medium, and were then incubated with equal amounts of the three AT preparations prior to the preperation of cell lysates and detection of bound AT by immunoblotting. The keratinocytes apparently retained large amounts of the β-AT analog and none of the K125M mutant (Fig. 4A, lanes 1 and 3). Keratinocytes retained only low levels of α-AT (Fig. 4A, lane 2). Taken together, these data suggest that the heparin binding affinity of AT plays an important role in its retention on the cell surface.

While the cellular retention of AT did not apparently alter matriptase activation and inhibition by HAI-1 (Fig. 4B, Total Matriptase, lanes 1, 2, and 3), the formation of matriptase-AT complex in the cells (Fig. 4B, AT, lanes 1, 2, and 3) and its subsequent shedding (Fig. 4B, Total MTP and AT, lanes 4, 5, and 6) were closely correlated with the cellular retention of AT (Fig. 4A). More importantly, the levels of shed free active matriptase was inversely correlated with the AT cellular retention (Fig. 4C). These data suggest that AT through binding to the cell surface can regulate the levels of shed active matriptase.

### Extracellular active matriptase can shed syndecan-1

**Figure 5:**
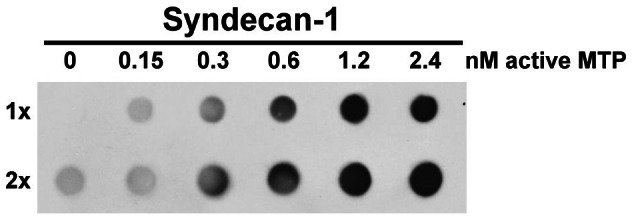
Syndecan-1 can be robustly shed by active matriptase. HaCaT cells were incubated with increasing active matriptase, as indicated, at 37°C for 30 min. The conditioned media were collected and subjected to dot blot analysis with two different loaded volumes (1× and 2×) for syndecan-1. Data are representative of three independent experiments done under similar conditions.

Given that cell surface HSPGs serve as the binding sites for AT, the rapid inhibition of active matriptase by AT suggests that matriptase activation might take place in close proximity to HSPGs. While tissue inhibitor of matrix metalloprotease (TIMP)-3-sensitive matrix metalloproteases (MMPs), such as MMP-7, plays a major role in the ectodomain shedding of syndecans 1 and 4, previous studies have also shown that syndecans 1 and 4 can be shed by trypsin-like serine proteases, such as plasmin [37], [38]. Given that matriptase exhibits potent trypsin-like activity and is apparently in close proximity to cell surface HSPGs, we wondered if matriptase might be able to mediate syndecan shedding. To test this hypothesis, we added purified active matriptase to human keratinocyte cultures and examined the level of syndecan-1 shedding. As shown in Fig. 5, the shedding of syndecan-1 was indeed stimulated by the addition of active matriptase in a dose-dependent manner. It is worthwhile noting that matriptase appears to be a much more potent protease with respect to the induction of syndecan 1 shedding than the other serine proteases and metalloprotease, reported to have this activity such as plasmin and MMP-7. Robust syndecan-1 shedding was induced with much lower concentrations of matriptase, and occurred much more rapidly, than has been reported for the other enzymes: 1 nM matriptase versus 60.24 µM for plasmin and 50 nM for MMP-7, with an incubation time of 30 min for matriptase versus 16 h for plasmin and 30 min for MMP-7 [37], [38].

## Discussion

Previous studies have revealed that matriptase activity is inhibited by two different types of serine protease inhibitor: the membrane-bound, Kunitz type inhibitor HAI-1 and the secreted serpin type inhibitor AT. Inhibition of matriptase by HAI-1 and AT are physiologically relevant events and not some culture system-induced artifact since both matriptase-HAI-1 and matriptase-AT complexes were initially purified and identified from human milk [39]. It has, however, been a mystery how matriptase-expressing cells employ AT as a physiological inhibitor of matriptase given that most of these cells express HAI-1 at a considerable molar excess compared to matriptase [10]. In the current study, our results indicate that the two distinct matriptase inhibitors are apparently involved in the regulation of two different types of substrate processing by matriptase: HAI-1 for prostasin activation and AT for the activation of HGF, acceleration of plasminogen activation, and shedding of syndecan-1. In addition to the rapid inhibition of nascent active matriptase that is mediated by both protease inhibitors, there are several important features and differences in the nature of matriptase inhibition that occurs. Both AT and HAI-1 inhibit matriptase on the cell surface but AT is also responsible for regulating the levels of free active matriptase that is shed into the extracellular milieu. With a relatively prolonged lifespan, extracellular active matriptase has the ability to activate and process secreted substrates, such as pro-uPA and pro-HGF. Extracellular active matriptase may also act on substrates anchored on the cell surface, mediating such processes as the shedding of HSPGs (Fig. 5) and activation of protease activated receptor 2 (PAR2) [40]. In contrast to extracellular active matriptase, cellular active matriptase is a short-lived species, evidence for which is the lack of cellular matriptase proteolytic activity detected in the cells in which robust matriptase activation has been induced. The short lifespan of cellular free active matriptase is the consequence of the very high molar ratio of HAI-1 to matriptase present in the cells [10], the potency of HAI-1 inhibitory activity against matriptase [41], and the co-localization of HAI-1 with matriptase on the cell surface and in the secretory pathway [11]. Prostasin is apparently the only *in vivo* substrate identified so far for the cellular active matriptase in keratinocytes [6], [9]. Although cellular active matriptase may also act on other substrates, loss of prostasin activation is likely to be responsible for most of the epidermal defects associated with matriptase deficiency in rodents since loss of prostasin phenocopies loss of matriptase. Therefore, the role of HAI-1 in matriptase inhibition is predominantly linked to prostasin activation in this system. In contrast, AT appears to have no role in the regulation of prostasin activation. The most interesting feature is that HAI-1 is responsible for a temporal and spatial control mechanism to ensure the proteolytic activities of matriptase and prostasin target a limited number of substrates without inappropriately cleaving other cellular proteins.

**Figure 6:**
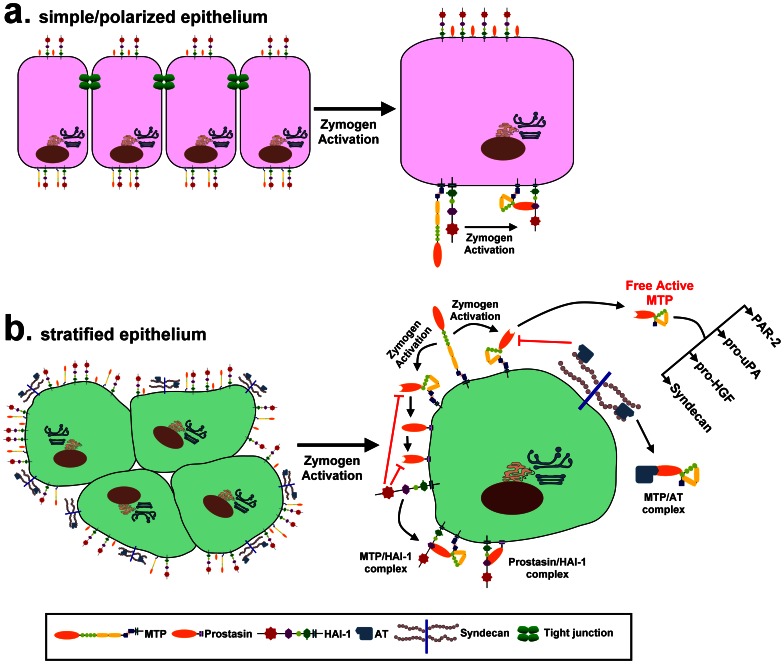
A schematic model of the different functional and regulatory mechanisms for matriptase control and activity that relate to the histological differences between simple/polarized and stratified epithelium. The subcellular distribution of matriptase (MTP), prostasin, and HAI-1 and the events following matriptase activation in the simple/polarized and the stratified epithelium are presented schematically. In simple/polarized epithelium, matriptase is targeted to the basolateral surface and prostasin is targeted to the apical surface with HAI-1 targeted to the both cell surface subdomains. The polarized expression of matriptase and prostasin is, however, likely not present in stratified epithelium, and as a result, matriptase gains an access to prostasin. When high level matriptase activation is induced, active matriptase is rapidly inhibited by nearby HAI-1 and the short-lived active matriptase can act on prostasin only in keratinocytes and not in polarized epithelial cells, due to the differential subcellular distributions of matriptase and prostasin between the two epithelia. Active prostasin is also rapidly inhibited by HAI-1 by forming a complex. A proportion of the active matriptase is shed from cell surface, an event that is evident in the stratified epithelial cells, and not in polarized epithelial cells. The shedding of active matriptase, the inhibition of active matriptase by AT bound to the cell via surface heparan sulfate proteoglycans, such as syndecans, is more obvious and important in stratified epithelial cells than in polarized epithelial cells. The active matriptase that escapes inactivation by HAI-1 or AT, can then act on its substrates, such as PAR-2, pro-uPA, pro-HGF, and syndecans.

While AT is commonly employed by epithelial cells to control matriptase activity [31], [39], keratinocytes are apparently unique among epithelial cells in terms of the extent to which AT contributes to matriptase inhibition. As shown in Fig. 3A, the ratio of matriptase-AT complex to matriptase-HAI-1 complex in both cell lysates and what is shed from the cells is vanishingly small in mammary and prostate epithelial cells. Furthermore, the vast majority of the cellular 110-kDa matriptase complex detected in 184 A1N4 cells is, in fact, matriptase complexed with protease nexin-1, and matriptase-AT complexes reprensent a minor species in the 110-kDa bands in mammary epithelial cells (Lin et al, unpublished observation). In contrast, although the matriptase-AT:matriptase-HAI-1 ratio in HaCaT keratinocytes varies somewhat from experiment to experiment, the ratio is estimated to be around 1∶2 (Fig. 3A) or 1∶3 (Fig. 3B). The greater role that AT plays in the control of matriptase activity in keratinocytes is likely a consequence of differencies in the functional and regulatory requirements for the roles that matriptase must fulfill in stratified versus simple/polarized epithelium, which are histologically and functionally distinct. First, both simple/polarized and stratified epithelial cells express matriptase and employ HAI-1 as an essential and indispensible partner. The close partnership between matriptase and HAI-1 is evident not only by their widespread co-expression in epithelial tissues, co-localization in the secretory pathway, and on cell surface subdomains, but also by the functional relationship that goes beyond their simple protease-antiprotease interaction. HAI-1 has been shown to participate in matriptase synthesis, intracellular traffincking and zymogen activation [11]. This unusual relationship is highlighted by the observation that the various defects associated with HAI-1 deletion in several animal models are rescued by the simultaneous deletion of matriptase [42], [43]. Secondly, it is likely that prostasin is a more important and physiologically relevant matriptase substrate in stratfied epithelium than in simple/polarized epithelium. In spite of their widespread co-expression in many epithelial tissues [44], matriptase is targeted to the basolateral plasma membrane [45] whereas prostasin is targeted to the apical plasma membrane in polarized epithelial cells [46]. Although matriptase could activate prostasin during the intracellular trafficking prior to their arrival at different subdomains of the cell surface of polarized epithelial cells [47], this mechanism would not likely be improtant due to the fact that both proteases are in their zymogen forms upon arrival at the cell surface. In contrast, stratified epithelial cells do not show the typical cell surface polarizartion with the result that matripase and prostasin are not spacially separated in the same way. This histological modification and adaption in stratified epithelium likely brings matriptase and prostasin together resulting in the keratinocyte-selective partnership between matriptase and prostasin. Thirdly, since HAI-1 must simultaneously act to inhibit both matriptase and prostasin, the activation of which occur at essentially the same time, there is competition for the pool of HAI-1 available to act on matriptase in keratinocytes which does not occur in polarized epithelial cells in which the HAI-1:matriptase ratio can be 10 or higher [10]. Thus, the reduced availibility of HAI-1 to inhibit matriptase might result in the increased reliance of keratinocytes on AT to control matriptase activity than in polarized epithelial cells. While less significant, in polarized epithelial cells, AT is known as a mechanism to control matriptase [31]. The enhanced role of AT in the regulation of matriptase in keratinocytes is likely an inevitable consequence of the histological nature of straitified epithelium. Finally, the availibility of AT on the cell surface depends on cell surface HSPGs and the binidng affinity of the AT to the HSPGs. This might represent another keratinocyte-selective mechanism by which the levels of active matriptase that is shed can be controlled by the levels of cell surface AT. In Fig. 6, we have constructed a model to summarize the different functional and regulatory mechanisms for matriptase control and activity that relate to the histological differences between simple/polarized and stratified epithelium.

Both the AT-mediated control of the level of active matriptase shed, and the events facilitated by the shed active matriptase (including accelerated plasmin generation, HGF activation, and shedding of syndecan 1), suggest that matriptase-mediated pericellular proteolysis might be very important in the context of wounding. Wound healing is a complex process involving tightly controlled pericellular proteolysis which is required for the clearance of fibrin clot and tissue debris, and the mobilization and activation of growth factors. At the time of wounding and during the course of subsequent wound healing, the activation of matriptase might be triggered by multiple mechanisms including the influx of blood during the acute phases of wounding (Chen and Lin, unpublished observation), by the elaboration of reactive oxygen species associated with inflammatory processes [7] and by tissue hypoxia during the process of healing. The resultant shed active matriptase can (as we have shown above) accelerate the generation of plasmin, the prominent protease for fibrinolysis, in wound healing. Plasmin generation is a membrane-associated event which requires both uPA and plasminogen bound to the surface of cells via the membrane receptor uPAR for uPA, and through less characterized membrane binding sites for plasminogen. The observation that both the shed active matriptase, and the cells from which it was shed are required in order for the generation of plasmin to be accelerated by matriptase activation, suggests that the active matriptase must interact with the cell surface in order to accelerate plasmin generation. This is consistent with what is known about the membrane-associated nature of plasmin generation. HGF has been shown to regulate several major processes in wound healing, including inflammation, angiogenesis, and re-epithelialization. The activation of HGF by shed active matriptase in the pericellular milieu further support a role for matriptase in wound repair. Matriptase may also contribute to wound healing by causing the shedding syndecan 1. The conversion of membrane-bound syndecans into their soluble forms can serve to down-regulate the signals transduced by syndecan-1 and thereby promote cell migration and facilitate wound closure [48]. Furthermore, the shed syndecans can bind to elastase and cathepsin G, the two major proteases secreted by neutrophils migrating to the injury site, thereby protecting these proteases from inhibition by serpin inhibitors present in the plasma [49]. The ability of cell surface associated AT to regulate the amount of free active matriptase shed from the cells provides a potential mechanism for keratinocytes to regulate matriptase-initiated pericellular proteolysis during wound healing in a controllable manner. Interestingly, much of the inhibition of matriptase by AT, appears to involve β-AT, and not α-AT. This might be attributed to the fact that β-AT has a higher affinity for heparin and heparin-like glycosaminoglycans than α-AT [50]. The differential role played between the two AT isoforms has also been seen in the inhibition by AT of thrombin coagulant activity in the injured vessel wall, in which β-AT is much more active than α-AT [51]. Since AT is able to rapidly bind to keratinocytes, those keratinocytes exposed to the blood in wounded skin would rapidly become coated with the serpin inhibitor. This cell surface-bound AT may then play an important role in the suppression of the high levels of free active matriptase that is induced by the influx of blood. This blood-driven matriptase activation and inhibition would provide keratinocytes with the ability to precisely control cell surface proteolysis without undesired damage during wound healing.

## Supporting Information

Figure S1 **The 110-kDa matriptase species is a protease/serpin complex.** *A.* HaCaT cells were induced to activate matriptase by a pH 6.0 buffer exposure. The conditioned buffer was collected and analyzed by immunoblot for matriptase using two different matriptase antibodies under either non-reducing and non-boiled conditions (NRNB), non-reducing and boiled conditions (NR, B), or reducing and boiled conditions (R, B). The antibodies used were mAb M24 that can detect both the zymogen and activated forms of matriptase (Total MTP) and a commercial polyclonal antibody, ST14, which recognizes the serine protease domain of matriptase and can be used to detect matriptase/serpin complexes under reducing conditions. *B.* A schematic model to show the fate of matriptase-serpin complexes after heating or chemical reduction. The shed matriptase-serpin complex (MTP/AT complex 110-kDa; the serpin is AT) remains intact after heating since the protease forms a covalent linkage with the serpin and so is resistant to the heat treatment. This covalent linkage between matriptase and the putative serpin cannot be dissociated by incubating the complexes with reducing agents, however, the disulfide bond that links the serine protease domain and non-catalytic domains of activated matriptase is disrupted by reducing agents. As a result, the complex was dissociated into the 45 kDa matriptase non-catalytic domain and a 95-kDa complex of matriptase serine protease domain with the serpin. **Caption**: We characterized the 110-kDa matriptase complex by comparing its migration in SDS polyacrylamide gel electrophoresis after heating the complex in the presence and absence of a reducing agent dithiothreitol. After heating, the migration of the complex was decreased slightly (Fig. S1A, comparing lanes 2 with lanes 1), suggesting that the interaction between matriptase and its binding protein is covalent and resistant to heating. After chemical reduction (Fig. S1A, lanes 3) that breaks the disulfide linkage holding the non-catalytic domains and serine protease domain of matriptase together, and destroys the epitope recognized by the matriptase mAb M24 (Fig. S1A, left panel, lane 3), the complex was dissociated and a protein band with a size around 95-kDa was detected by a matriptase antibody directed against the serine protease domain of matriptase (Fig. S1A, right panel, lane 3). These data suggest that the matriptase molecule in the complex is in an activated form and interacts with the binding protein via a covalent bond.
(DOCX)

Figure S2 **Identification of AT as a component of the novel 110-kDa matriptase complex.** The protein bands indicated by *a* and *b* in Fig. 3C, left panel, were subjected to protein identification by digestion with trypsin and analysis by MS/MS. Among the tryptic peptides obtained from protein band *a*, 10 peptides matched to matriptase and 18 peptides matched to AT. Ten peptides obtained from protein band *b* matched to matriptase and 20 matched to AT. These amino acid sequences are presented using a single letter with their position in the full sequence indicated with numbers at the beginning and the end of each peptide. **Caption**: The partial sequences generated from the purified matriptase 110-kDa complex are presented in Figure S2. The sequence information confirms that the purified protein bands contain matriptase and indicates that the matriptase binding protein present in the 110-kDa complex is bovine antithrombin (AT).
(DOCX)
