# Supplementary figures and images for: Antithrombin Regulates Matriptase Activity Involved in Plasmin Generation, Syndecan Shedding, and HGF Activation in Keratinocytes
Source: PLoS One. 2013 May 13;8(5):e62826. doi: 10.1371/journal.pone.0062826 (PMC3652837; doi:10.1371/journal.pone.0062826)

**Figure S1**


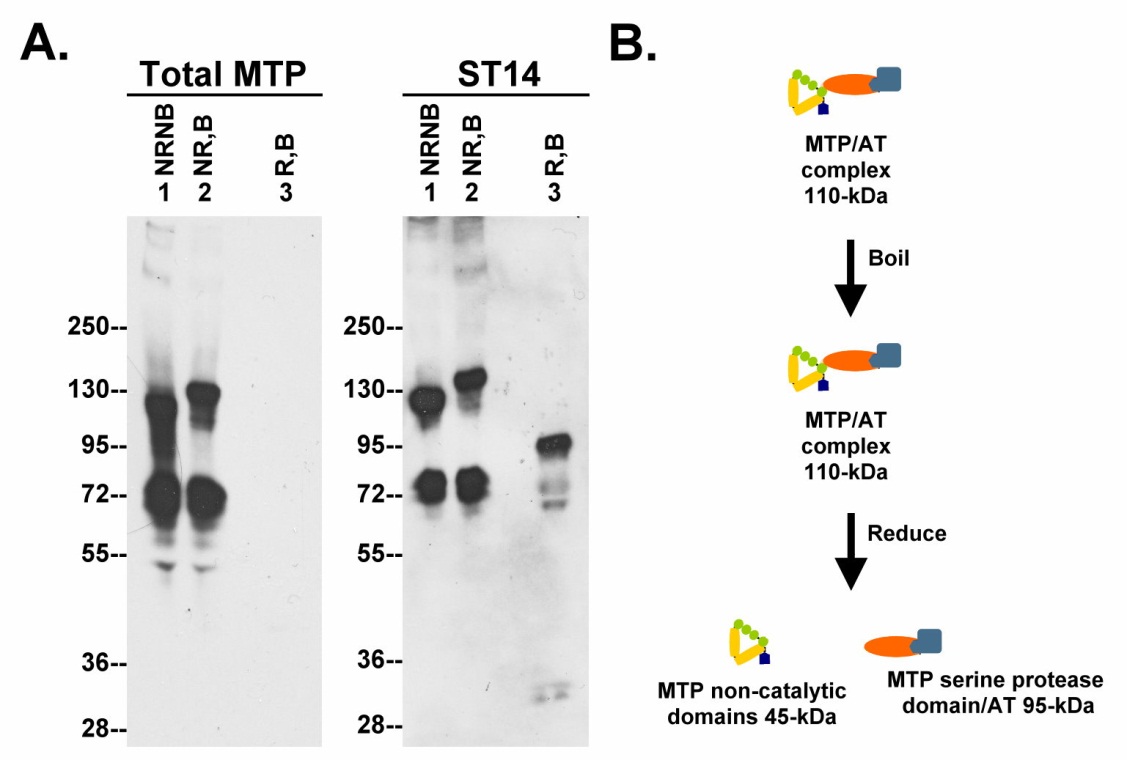

Supplement: Figure S1 — The 110-kDa matriptase species is a protease/serpin complex. A. HaCaT cells were induced to activate matriptase by a pH 6.0 buffer exposure. The conditioned buffer was collected and analyzed by immunoblot for matriptase using two different matriptase antibodies under either non-reducing and non-boiled conditions (NRNB), non-reducing and boiled conditions (NR, B), or reducing and boiled conditions (R, B). The antibodies used were mAb M24 that can detect both the zymogen and activated forms of matriptase (Total MTP) and a commercial polyclonal antibody, ST14, which recognizes the serine protease domain of matriptase and can be used to detect matriptase/serpin complexes under reducing conditions. B. A schematic model to show the fate of matriptase-serpin complexes after heating or chemical reduction. The shed matriptase-serpin complex (MTP/AT complex 110-kDa; the serpin is AT) remains intact after heating since the protease forms a covalent linkage with the serpin and so is resistant to the heat treatment. This covalent linkage between matriptase and the putative serpin cannot be dissociated by incubating the complexes with reducing agents, however, the disulfide bond that links the serine protease domain and non-catalytic domains of activated matriptase is disrupted by reducing agents. As a result, the complex was dissociated into the 45 kDa matriptase non-catalytic domain and a 95-kDa complex of matriptase serine protease domain with the serpin. Caption: We characterized the 110-kDa matriptase complex by comparing its migration in SDS polyacrylamide gel electrophoresis after heating the complex in the presence and absence of a reducing agent dithiothreitol. After heating, the migration of the complex was decreased slightly (Fig. S1A, comparing lanes 2 with lanes 1), suggesting that the interaction between matriptase and its binding protein is covalent and resistant to heating. After chemical reduction (Fig. S1A, lanes 3) that breaks the disulfide linkage holding the [file pone.0062826.s001.docx]

**Figure S2**


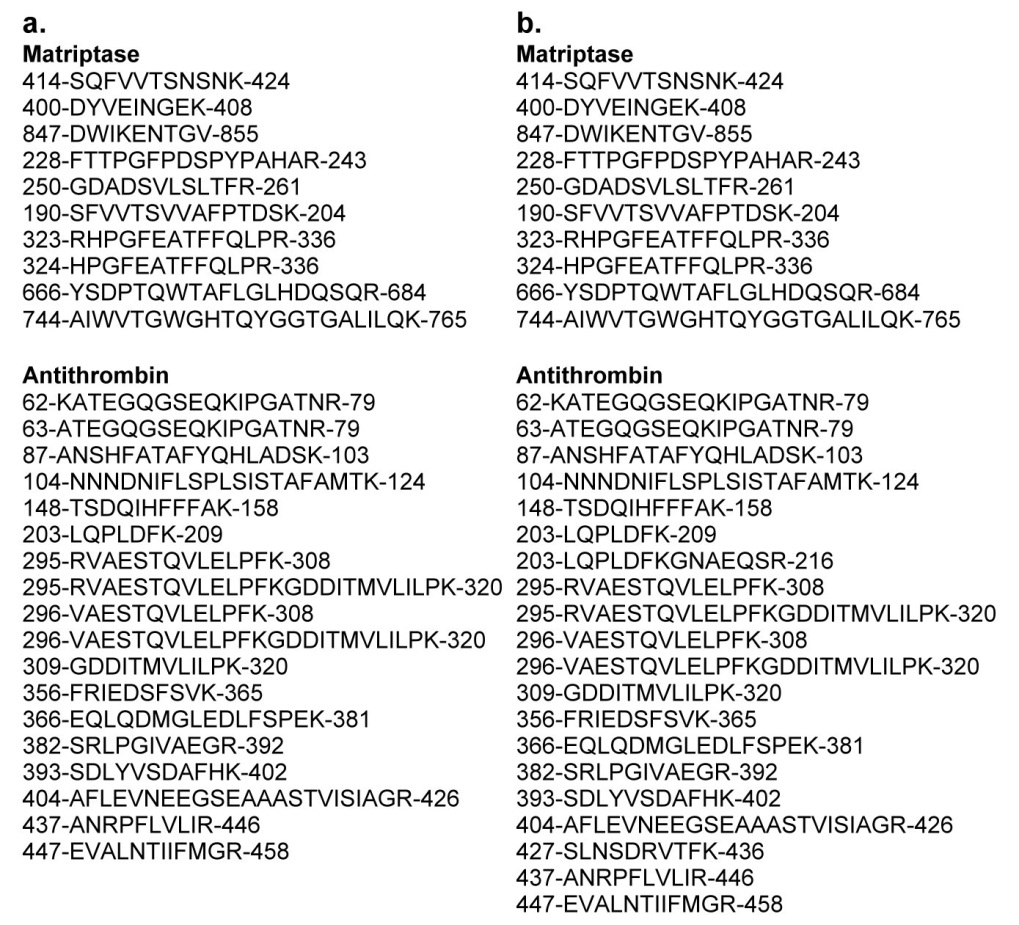

Supplement: Figure S2 — Identification of AT as a component of the novel 110-kDa matriptase complex. The protein bands indicated by a and b in Fig. 3C, left panel, were subjected to protein identification by digestion with trypsin and analysis by MS/MS. Among the tryptic peptides obtained from protein band a, 10 peptides matched to matriptase and 18 peptides matched to AT. Ten peptides obtained from protein band b matched to matriptase and 20 matched to AT. These amino acid sequences are presented using a single letter with their position in the full sequence indicated with numbers at the beginning and the end of each peptide. Caption: The partial sequences generated from the purified matriptase 110-kDa complex are presented in Figure S2. The sequence information confirms that the purified protein bands contain matriptase and indicates that the matriptase binding protein present in the 110-kDa complex is bovine antithrombin (AT). (DOCX) [file pone.0062826.s002.docx]
